# Novel Insights Into the Causal Association Between Dietary Factors and Risk of Urinary Calculus: A Multivariate and Two‐Step Mendelian Randomization Analysis

**DOI:** 10.1002/fsn3.70958

**Published:** 2025-09-12

**Authors:** Xinling Tang, Youjia Qiu, Bingyi Song, Yuchen Tao, Ziqian Yin, Menghan Wang, Na Ji, Zhouqing Chen, Zhong Wang, Xuedong Wei

**Affiliations:** ^1^ Department of Urology Surgery The First Affiliated Hospital of Soochow University Suzhou Jiangsu Province China; ^2^ Department of Neurosurgery The First Affiliated Hospital of Soochow University Suzhou Jiangsu Province China; ^3^ Department of Neurology The First Affiliated Hospital of Soochow University Suzhou Jiangsu Province China

**Keywords:** blood metabolites, dietary factors, Mendelian randomization analysis, urinary calculus

## Abstract

Previous observational studies preliminarily unveiled dietary habits that are associated with urinary calculus (UC). However, the causal association remained unclear. This study employed two‐sample Mendelian randomization (MR) to assess the causal effects of dietary factors on UC using genome‐wide association data. Multivariable MR (MVMR) was applied to identify independent dietary influences, and two‐step MR explored mediation by 30 common biomarkers. Genetically predicted intake of alcohol, coffee (including decaffeinated, instant, and ground coffee), psychoactive drinks, tea, fruits (including dried fruit), and preferences for coffee without sugar, fruit, and white wine were inversely associated with calculus of the kidney and ureter (all *p* < 0.05). Additionally, preferences for fruit (including cherry and plum) and coffee with sugar were inversely associated with lower urinary tract stones (*p* < 0.05). MVMR confirmed that the protective effects of coffee (*p* = 0.0008), instant coffee (*p* = 0.0003), psychoactive drinks (*p* = 0.014), fruit (fruit consumption *p* = 4.55 × 10^−5^, fruit liking *p* = 0.0007), and dried fruit (*p* = 0.003) on calculus of the kidney and ureter, as well as cherry (*p* = 0.001), fruit (*p* = 0.005), and plum liking (*p* = 0.0001) on calculus of the lower urinary tract, were independent of other dietary factors. Two‐step MR further suggested calcium partially mediated the effects of fruit (4.9%), dried fruit (8.8%), and cherry liking (2.7%) on UC. These findings provide genetic evidence for the protective roles of specific dietary factors in UC development and highlight potential biological mediators, offering new insights for prevention strategies.

## Graphical abstract

This study investigated potential causal associations between dietary habits, biomarkers, and urinary calculus (UC). Causal influence of coffee consumption, fruit liking, instant coffee consumption, psychoactive drinks consumption, dried fruit consumption, fruit consumption on calculus of the kidney and ureter were independent of other factors. Among promising biomarkers, calcium may be the mediator between the causal relationship between dietary habits and urinary calculus. Created in BioRender. Lu, D. (2025) https://BioRender.com/eh2j48r.

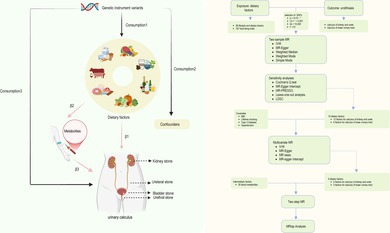

## 1 Introduction

Urinary Calculus (UC) is a common disease of urology, with high incidence and high recurrence rate. According to the different locations of the stones, they can be divided into kidney stones (KS), ureteral stones (US), bladder stones, and urethral stones. According to the latest epidemiology investigation, more than 115 million patients suffer from the burden of disease caused by KS worldwide (Zhang et al. 2022). In the United States, the prevalence of KS has reached 8.8%, with a relatively higher prevalence in men than in women (10.6% vs. 7.1%) (Hoffman et al. 2021). UC also demonstrates high recurrence rates among patients, and it has been estimated that approximately half of the patients may experience recurrence within 5–10 years after the first occurrence (Shastri et al. 2023). UC can lead to urinary tract obstruction and infection and is also a potential risk factor for other systemic diseases, such as diabetes mellitus, cardiovascular disease (Ferraro et al. 2013; Rule et al. 2010), bone fracture (Taylor et al. 2016), and chronic kidney disease (El‐Zoghby et al. 2012), which can seriously jeopardize the quality of life and bring a substantial financial burden to patients. Hence, effective prevention is critical for patients with UC.

A variety of potential factors may play a role in the formation of UC, such as metabolic disorders, genetic factors, anatomical variations, and functional abnormalities. According to previous literature, dietary factors have been proven to be associated with UC. Previous viewpoints suggested that dietary factors could affect urine risk profile and the supersaturation with the stone‐forming salt, thus increasing the risk of UC formation (Siener 2021; Siener et al. 2005). However, previous evidence linking dietary factors and UC comes from observational studies, which are limited by reverse causality and confounding factors. The causal relationship between dietary factors and UC remains unclear due to a lack of randomized controlled trial (RCT) data, and investigating this in RCTs is challenging due to accessibility and ethical constraints. To address the limitations of small sample sizes and confounding factors in traditional observational studies, we employed Mendelian randomization (MR) analysis to investigate causal effects. MR uses genetic variants, typically single‐nucleotide polymorphisms (SNPs) from genome‐wide association studies (GWAS), as instrumental variables (IVs) to explore causal associations between exposures and outcomes. To assess the role of dietary factors in UC formation, we conducted a comprehensive analysis using two‐sample MR, multivariable MR (MVMR), and two‐step MR methods, offering insights for prevention and therapeutic strategies.

## 2 Methods

### 2.1 Study Design

**FIGURE 1:**
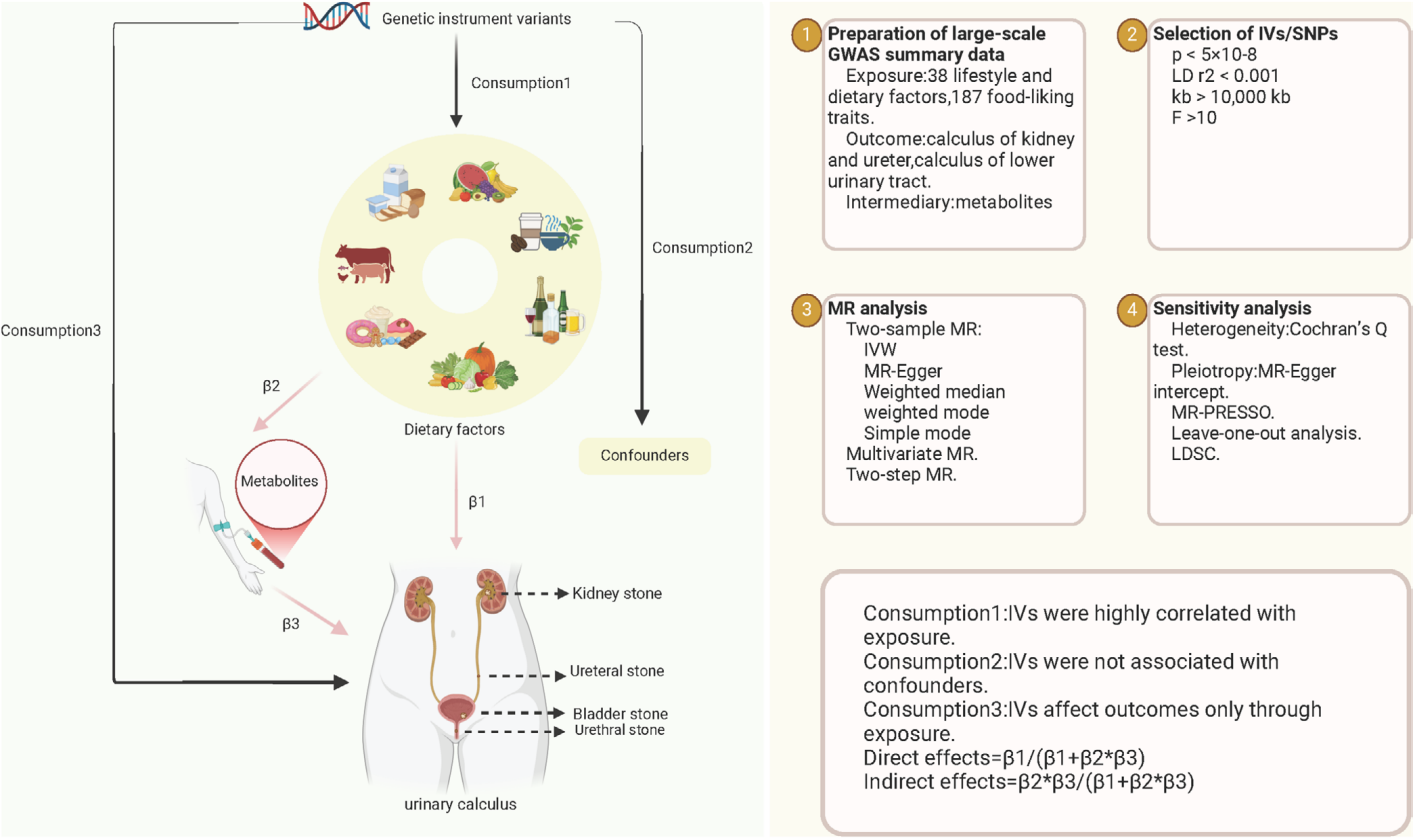
The overview of the MR analysis design. IV, instrumental variable; IVW, inverse‐variance weighted; MR, Mendelian randomization; MR‐PRESSO, MR pleiotropy residual sum and outlier; SNPs, single‐nucleotide polymorphisms. Created in BioRender. Lu, D. (2025) https://BioRender.com/eh2j48r.

UC was considered as the outcome, encompassing both calculus of the kidney and ureter, as well as calculus of the lower urinary tract. Considering that smoking, hypertension, type 2 diabetes, and body mass index (BMI) are common risk factors for stone formation, we included these variables along with the significant dietary exposures in the multivariable analysis to control for their potential confounding effects. Previous studies have demonstrated that elevated serum calcium levels may influence stone formation (Lovegrove et al. 2023). To further elucidate the mechanisms underlying the association between dietary factors and urolithiasis, we incorporated 30 metabolites into mediation analyses based on the positive findings from the MVMR results. The overall study design is provided in Figure 1.

The validity of MR estimates critically depends on satisfying three core instrumental variable assumptions: (1) Relevance assumption: Genetic instruments must demonstrate genome‐wide significant associations with target dietary exposures; (2) Independence assumption: Selected SNPs exhibit no pleiotropic associations with known UC confounders; (3) Exclusion restriction assumption: Genetic effects on UC risk operate exclusively through the specified dietary pathways, without direct biological interactions (Haycock et al. 2016).

This study was based on large GWAS and publicly available data from various groups and consortia, and informed consent was obtained from the participants for the original studies; therefore, no additional ethical approval was required for this study.

### 2.2 Data Source

For dietary factors, a recent GWAS study (Pirastu et al. 2022) has identified 38 lifestyle and dietary factors among 445,779 European participants, such as alcohol, coffee, beef, fruit, vegetables, and fish consumption (GCST90096892‐GCST90096929). Additionally, another large GWAS study (May‐Wilson et al. 2022) with 161,625 UK Biobank participants was also used as exposures, which included 187 food‐liking traits (GCST90094678–GCST90094873).

For outcome ascertainment, UC phenotypes were stratified into calculus of kidney and ureter (kidney/ureter, *N*‐cases =12,999; *N*‐controls = 486,185) and calculus of lower urinary tract (bladder/urethra, *N*‐cases = 1878; *N*‐controls = 48,6185) using FinnGen R12 registry data. Case definitions adhered to ICD‐10 code N20 (renal/ureteral calculus) and ICD‐8/9 code 592 (nephrolithiasis), with comprehensive endpoint specifications available in the FinnGen phenotype registry (FinnGen Data Freeze 12).

Data for lifetime smoking, hypertension, type 2 diabetes, and BMI were obtained from publicly available GWAS databases and recent publications; blood metabolite data were derived from the IEU OpenGWAS project. Complete variant‐to‐phenotype associations are accessible through Table S2.

### 2.3 Identification of IVs

In order to improve the accuracy of our results, we strictly screened the dietary factors GWAS data from the literature. We used the genome‐wide significant threshold *p* < 5 × 10^−8^ for screening to obtain more IVs for our analysis, and at the same time, we excluded SNPs that were significantly associated with the outcome (*p* > 5 × 10^−5^). Second, strict threshold clustering (*r* ^2^ < 0.001, kb > 10,000 kb) was used to ensure the independence of the included SNPs. Third, IVs highly associated with determined risk factors of calculi (such as BMI, smoking, hypertension, and type 2 diabetes) were excluded before MR analysis, and these factors related to the calculi were searched on the GWAS catalog website (https://www.ebi.ac.uk/gwas/). Finally, the *F*‐statistic of all included SNPs was over 10 to ensure the strength of IVs; the *F*‐statistic was calculated using the following formula (Huang et al. 2021).

$$F={\left(\frac{\beta }{\mathrm{SE}}\right)}^{2}$$

### 2.4 Statistical Analysis

Our causal inference framework employed a hierarchical analytical approach integrating multiple Mendelian randomization (MR) methodologies with summary‐level genome‐wide association study (GWAS) data. The inverse‐variance weighted (IVW) method served as the main statistical model for MR analysis (Burgess et al. 2013), providing asymptotically efficient causal estimates under the assumption of balanced horizontal pleiotropy. We further validated the results using four complementary MR methods: MR‐Egger provides reliable estimates even when all SNPs are invalid instruments (Bowden et al. 2015). The weighted median method yields consistent results if at least 50% of the instruments are valid (Bowden et al. 2016). The weighted mode improves robustness by averaging SNP effects (Hartwig et al. 2017). When only one instrument is available, the simple mode directly uses its effect estimate (Hemani, Zheng, et al. 2018). The odds ratio (OR) and its 95% confidence interval (95% CI) indicate a causal effect between the exposure and outcome. The *p* value was adjusted by the false discovery rate (FDR), which adjusts the results of multiple comparisons (*q*). A *p* < 0.05 and *q* < 0.1 indicated a significant causal association (Glickman et al. 2014).

### 2.5 Sensitivity Analyses

Sensitivity analysis was conducted to detect the robustness of the MR estimates. Cochran's *Q* test assessed the heterogeneity among genetic variants. MR‐PRESSO was utilized to determine potential outlier SNPs, while directional pleiotropy was estimated using the MR‐Egger intercept method (Hartwig et al. 2016; Hemani, Bowden, and Davey Smith 2018). The scatter plots depicted the association between dietary factors and calculus, and the slope represents estimated positive or negative causal effects. Forest plots present causal estimates obtained from each genetic variant, visualizing the heterogeneity of MR results. A funnel plot depicted the distribution of SNPs, and a symmetric distribution indicates no horizontal pleiotropy. Leave‐one‐out plots were performed multiple times of MR analysis to test whether individual outliers strongly drove causal effects by excluding one SNP in turn (Skrivankova et al. 2021).

To investigate the overall genetic correlation between dietary factors and urolithiasis, we employed Linkage Disequilibrium Score regression (LDSC) (Chen and He 2023) in our Mendelian Randomization analysis. LDSC is a well‐established method for estimating genetic correlations and heritability by analyzing the relationship between SNP effect sizes and their linkage disequilibrium (LD) scores. It calculates LD scores from reference data and regresses GWAS summary statistics on these scores to quantify shared genetic architecture between traits (Bulik‐Sullivan et al. 2015).

### 2.6 Multivariate MR and Two‐Step MR

To address potential horizontal pleiotropy, we used MVMR to assess the independent effects of significant dietary factors on UC after adjusting for relevant covariates. Unlike univariate MR, MVMR isolates the direct effect of each exposure; in this study, MVMR was further applied to adjust for key confounders, including hypertension, type 2 diabetes, BMI, and lifetime smoking, ensuring the observed associations were not driven by these factors.

To identify whether the observed dietary factors are related to calculus risk directly, we evaluated the relationship between dietary factors and common blood metabolites detected in the human body, such as urea, glucose, and calcium via two‐step MR analysis, which may be potential mediators. In two‐step MR, the impact of dietary factors on calculus after accounting for potential mediators is denoted as the direct effect, while the intermediary effect from potential mediators is considered the indirect effect. We considered the existence of a potential mediator when the following conditions were met: (1) dietary factors were correlated with calculus, with no requirement of adjusting mediators (*β*1); (2) dietary factors were correlated with the mediator (*β*2); and (3) the mediator was correlated with calculus (*β*3). The percentage of mediation was calculated with the product of coefficients by applying the following formula: (*β*2 × *β*3)/(*β*1) (Burgess et al. 2015).

### 2.7 MR lap Analysis

Since GWAS summary statistics of dietary factors and metabolites were derived from UKB, MR estimates could be affected by sample overlap and may lead to high false positives. We therefore introduced a novel method called MR lap to adjust IVW findings considering the potential bias from unmeasurable sample overlap (Mounier and Kutalik 2023). When the observed‐adjusted effect difference is nonsignificant (*p* > 0.05), IVW‐MR results remain reliable; however, a statistically significant disparity (*p* < 0.05) prioritizes the adjusted effect, which stays unaltered by sample overlap.

All analyses were performed using R 4.4.1. The MendelR package was utilized for Mendelian randomization and LDSC analyses, and data visualization was conducted using packages such as forestploter and ComplexHeatmap. The MRlap analysis was conducted based on the MRlapPro package.

## 3 Results

### 3.1 IVs Selection and Two Sample MR Results

SNPs selected as IVs for dietary factors and finally used to estimate the effects in MR analysis are shown in Tables S3 and S4. The *F* statistic of each SNP was more than 10.

**FIGURE 2:**
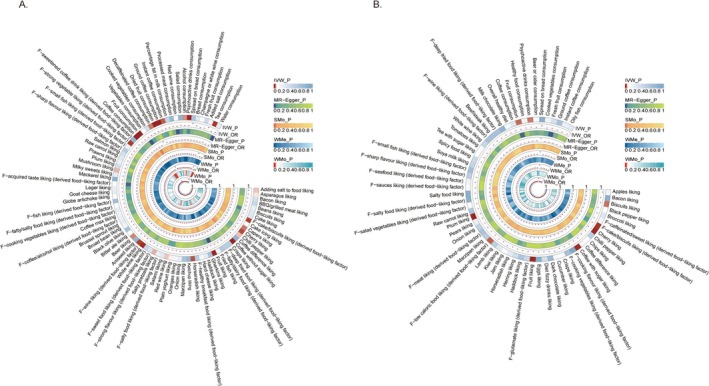
Heatmap of the MR estimates of dietary factors and UC. (A) Calculus of kidney and ureter; (B) calculus of lower urinary tract. The different colors represent the *p* values derived from the causality analysis using each method. IVW, inverse‐variance weighted method; OR, odds ratios; SMo, simple mode; WMe, weighted median; WMo, weighted mode.

**FIGURE 3:**
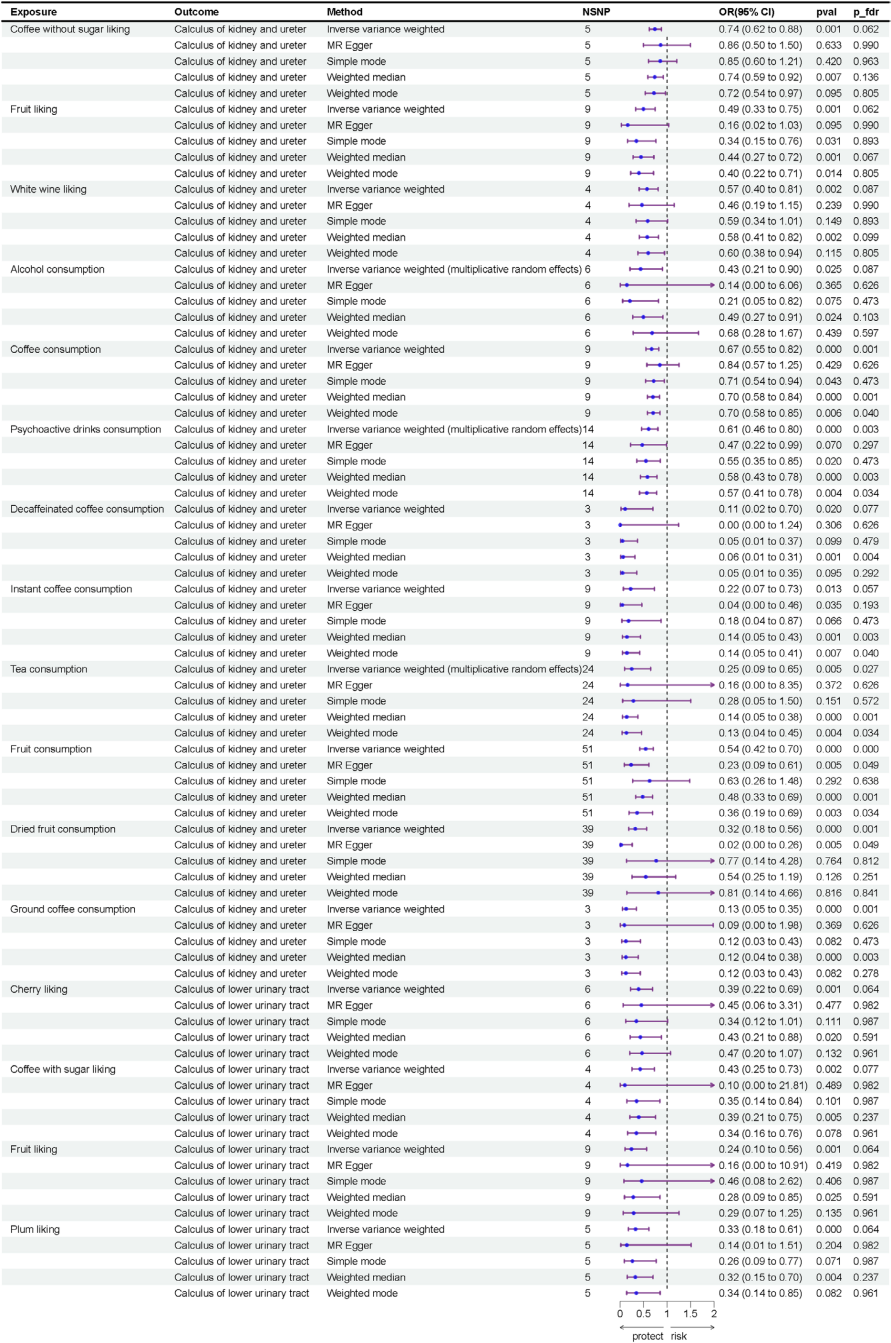
Result of two‐sample MR analysis on dietary factors and UC after FDR correction. IVW, inverse‐variance weighted method; OR, odds ratio.

Among 38 food consumptions, nine demonstrated statistical significance with the risk of calculus of kidney and ureter: alcohol consumption (OR = 0.43, 95% CI = 0.21–0.90, *p* = 0.025), coffee consumption (OR = 0.67, 95% CI = 0.54–0.82, *p* = 0.0001), psychoactive drinks consumption (OR = 0.61, 95% CI = 0.45–0.80, *p* = 0.0004), decaffeinated coffee consumption (OR = 0.11, 95% CI = 0.01–0.69, *p* = 0.020), instant coffee consumption (OR = 0.22, 95% CI = 0.07–0.73, *p* = 0.012), ground coffee consumption (OR = 0.13, 95% CI = 0.04–0.35, *p* = 5.421 × 10^−5^), tea consumption (OR = 0.25 95% CI = 0.09–0.65, *p* = 0.005), fruit consumption (OR = 0.54, 95% CI = 0.42–0.70, *p* = 4.340 × 10^−6^), and dried fruit consumption (OR = 0.32, 95% CI = 0.18–0.56, *p* = 7.305 × 10^−6^). Among the 187 food liking traits, coffee without sugar liking (OR = 0.74, 95% CI = 0.62–0.88, *p* = 0.0006), fruit liking (OR = 0.49, 95% CI = 0.33–0.75, *p* = 0.0008), and white wine liking (OR = 0.57, 95% CI = 0.40–0.81, *p* = 0.002) were negatively associated with the formation of calculus of kidney and ureter, while cherry liking (OR = 0.39, 95% CI = 0.22–0.69, *p* = 0.001), coffee with sugar liking (OR = 0.43, 95% CI = 0.25–0.73, *p* = 0.002), fruit liking (OR = 0.24, 95% CI = 0.10–0.56, *p* = 0.001), and plum liking (OR = 0.33, 95% CI = 0.18–0.61, *p* = 0.0005) were negatively associated with the formation of calculus of lower urinary tract. All the above results remained significant after FDR correction, with *q* < 0.1. The results are presented in Figures 2 and 3, with the complete findings available in Tables S7 and S8.

### 3.2 Sensitivity Analyses

Sensitivity analyses were also performed to assess the robustness of MR estimates. MR‐Egger intercept analyses indicated that, except for dried fruit consumption, no evidence of horizontal pleiotropy was observed for the other results (*p* > 0.05). Cochran's *Q* test indicated significant heterogeneity in the associations of alcohol consumption, psychoactive drinks consumption, and tea consumption with calculus of the kidney and ureter, while no heterogeneity was observed for the other results (*p* > 0.05) (see Table S9). In addition, scatter plots showed consistent direction of results across methods, leave‐one‐out analysis did not find any outlier SNPs, and the results of funnel plots and forest plots are shown in Figures S1–S8. In conclusion, the present study found a consistent and robust association between several dietary factors (including several different types of coffee, fruit and white wine) and UC. MR‐PRESSO did not identify any potential pleiotropy IVs in MR estimates (Tables S5 and S6).

After multiple adjustments, the LDSC analysis was conducted to evaluate the genetic correlation between dietary factors and UC, and it concluded that alcohol consumption (*r*_g_ = −0.09, SE = 0.05, *p* = 0.0488), coffee without sugar liking (*r*_g_ = −0.21, SE = 0.05, *p* = 8.36 × 10^−5^), dried fruit consumption (*r*_g_ = −0.15, SE = 0.04, *p* = 0.0001), fruit consumption (*r*_g_ = −0.17, SE = 0.04, *p* = 4.17 × 10–6), fruit liking (*r*_g_ = −0.16, SE = 0.05, *p* = 0.003), and tea consumption (*r*_g_ = −0.21, SE = 0.05, *p* = 4.23 × 10–6) showed a strong genetic correlation with calculus of the kidney and ureter, while coffee with sugar liking (*r*_g_ = −0.2, SE = 0.08, *p* = 0.0085) showed a strong genetic correlation with calculus of the lower urinary tract (Table 1).

**TABLE 1:** Result of LDSC of significant dietary factors and UC.

Exposure	Outcome	rg	rg_se	rg_p
Alcohol consumption	Calculus of kidney and ureter	−0.095	0.048	0.049
Coffee consumption	Calculus of kidney and ureter	−0.025	0.050	0.627
Coffee without sugar liking	Calculus of kidney and ureter	−0.210	0.053	8.36 × 10^−5^
Decaffeinated coffee consumption	Calculus of kidney and ureter	−0.116	0.079	0.141
dried fruit consumption	Calculus of kidney and ureter	−0.150	0.039	1.09 × 10^−4^
fruit consumption	Calculus of kidney and ureter	−0.166	0.036	4.17 × 10^−6^
Fruit liking	Calculus of kidney and ureter	−0.156	0.053	0.003
ground coffee consumption	Calculus of kidney and ureter	−0.030	0.074	0.691
instant coffee consumption	Calculus of kidney and ureter	0.067	0.052	0.202
Psychoactive drinks consumption	Calculus of kidney and ureter	−0.078	0.045	0.085
tea consumption	Calculus of kidney and ureter	−0.208	0.045	4.23 × 10^−6^
White wine liking	Calculus of kidney and ureter	−0.034	0.055	0.538
Cherry liking	Calculus of lower urinary tract	−0.095	0.093	0.307
Coffee with sugar liking	Calculus of lower urinary tract	−0.204	0.097	0.035
Fruit liking	Calculus of lower urinary tract	−0.107	0.094	0.252
Plum liking	Calculus of lower urinary tract	0.021	0.097	0.826

### 3.3 MVMR And Two‐Step MR

**FIGURE 4:**
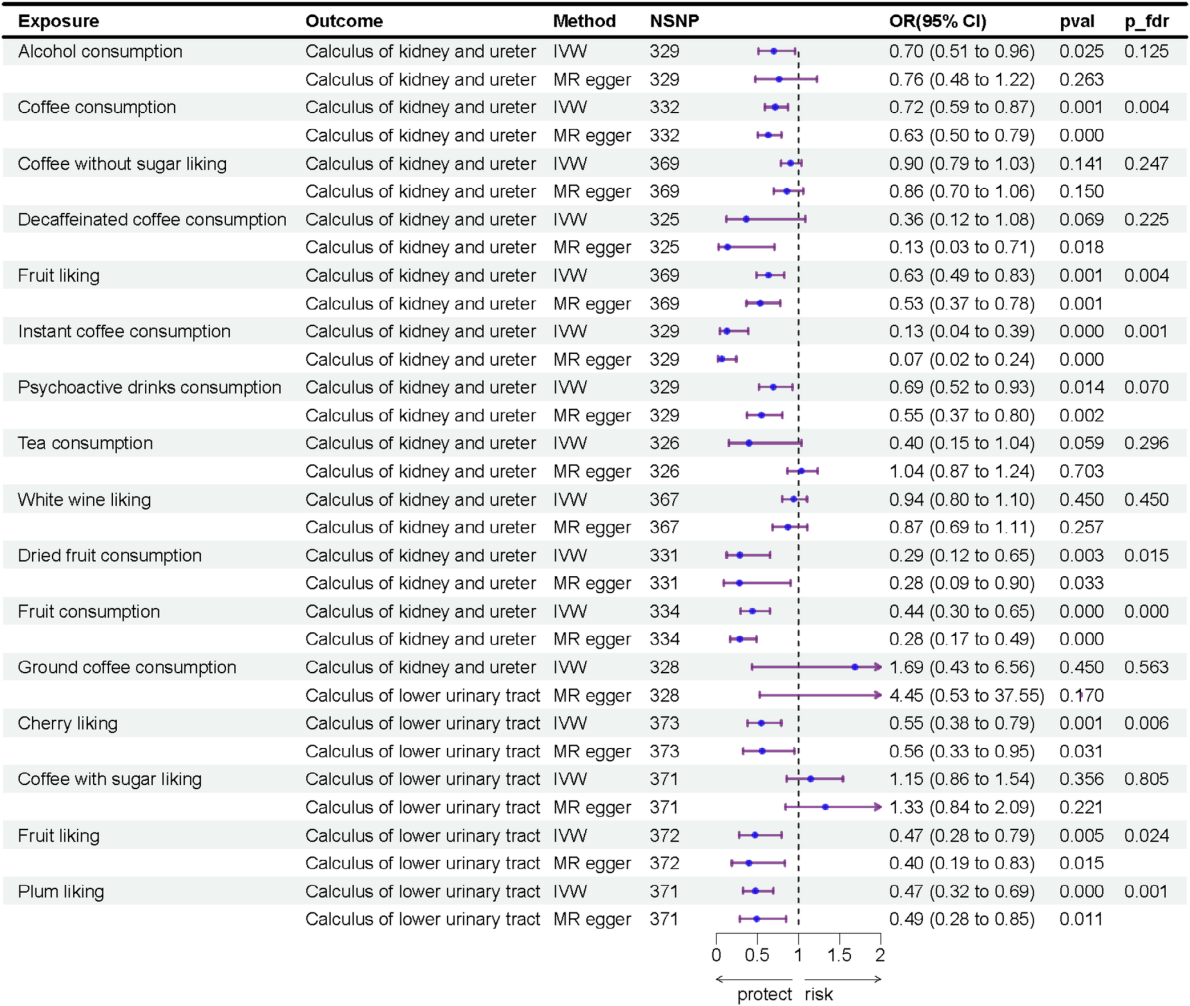
Result of multivariate MR on significant dietary factors and UC.

MVMR analysis indicates that genetically predicted coffee consumption (OR = 0.72, 95% CI = 0.59–0.87, *p* = 0.0008), fruit liking (OR = 0.63, 95% CI = 0.49–0.83, *p* = 0.0007), instant coffee consumption (OR = 0.13, 95% CI = 0.04–0.39, *p* = 0.0003), psychoactive drinks consumption (OR = 0.69, 95% CI = 0.51–0.93, *p* = 0.014), dried fruit consumption (OR = 0.29, 95% CI = 0.12–0.65, *p* = 0.003) and fruit consumption (OR = 0.44, 95% CI = 0.30–0.65, *p* = 4.55 × 10^−5^) exhibited robust inverse associations with calculus of kidney and ureter, independent of other factors, while cherry liking (OR = 0.54, 95% CI = 0.38–0.79, *p* = 0.001), fruit liking (OR = 0.47, 95% CI = 0.28–0.79, *p* = 0.005) and plum liking (OR = 0.47, 95% CI = 0.32–0.69, *p* = 0.0001) showed a causal association with calculus of lower urinary tract independent of other factors. The results are shown in Figure 4, and detailed data are provided in Table S10.

The proportion of mediation effect in two‐step MR is shown in Tables S11–S15. After multiple corrections, calcium showed a partial mediating effect in several pathways, including the associations between fruit (4.9%), dried fruit (8.8%) and calculus of the kidney and ureter. In addition, the suggestive intermediary effect of calcium was found on the causal association between cherry liking and calculus of the lower urinary tract (2.7%) (Table S15). The results of the Steiger directionality test indicated no evidence of reverse causality. Detailed results are shown in Table 2.

**TABLE 2:** Result of Two‐step MR of significant dietary factors and UC.

Exposure	Outcome	Intermediary factors	Method	Mediation proportion	lci95	uci95	*p*
Fruit consumption	Calculus of kidney and ureter	Calcium	IVW	0.050	0.012	0.087	0.009
Dried fruit consumption	Calculus of kidney and ureter	Calcium	IVW	0.088	0.039	0.138	4.210 × 10^−4^
Cherry liking	Calculus of lower urinary tract	Calcium	IVW	0.027	0.002	0.052	0.030

### 3.4 MRlap Analysis

The results of the MRlap analysis are shown in Table S16. The IVW method's robustness is confirmed by the fact that the data corrected by MRlap agreed with the findings of the initial MR analysis. Similarly, it was shown that sample overlap had no effect on the causal connection between dietary factors and kidney stones.

## 4 Discussion

This study is grounded in large‐scale GWAS data and through establishing MR analysis, a total of 38 dietary consumptions and 187 diet‐related traits were incorporated. It systematically and comprehensively explored the potential causal associations between different dietary factors and UC. Sixteen protective associations were identified, including twelve for calculus of kidney and ureter and four for calculus of lower urinary tract. Fruit, dried fruit, various types of coffee, tea, and white wine were negatively associated with upper UC, while coffee with sugar, cherry, fruit, and plum liking were protective against lower UC. The above results were significant after FDR correction with *q* < 0.1. Calcium partially mediated the effects of fruit, dried fruit, and cherry on UC risk.

Coffee and tea are among the most commonly consumed beverages, and a number of past reviews and large cohort studies (Geng et al. 2022; Littlejohns et al. 2020) have generally supported the potential preventive effects of coffee and tea on kidney stones. Caffeine, a key component of regular coffee, antagonizes renal tubular adenosine A_1_/A_2_ receptors, reducing sodium and water reabsorption to increase urine output (Rieg et al. 2005), thereby lowering urinary supersaturation of calcium and oxalate. Coffee may reduce urinary calcium and uric acid excretion while increasing citrate and magnesium levels. Citrate forms soluble complexes with calcium, and magnesium competitively binds oxalate, together inhibiting crystal precipitation (Peerapen and Thongboonkerd 2016; Phillips et al. 2015). Unlike caffeinated coffee, the protective effects of decaffeinated coffee primarily stem from antioxidant and anti‐inflammatory pathways centered on retained bioactive compounds including polyphenols and diterpenoids, rather than diuresis‐dependent mechanisms. As a principal bioactive constituent, chlorogenic acid (CGA) mediates protective effects through modulation of urinary ion homeostasis and crystallization inhibitors, specifically by reducing calcium bioavailability and enhancing citrate complexation (Tajik et al. 2017). It has been reported that CGA can exert anti‐inflammatory and antioxidant effects by attenuating various signaling pathways activated by pathogens, such as NF‐κB, C‐JNK, extracellular signal‐regulated kinases (ERK), and by inhibiting multiple pro‐inflammatory factors like TNF‐α, IL‐1β, etc. (Hwang et al. 2014; Ji et al. 2013; Shan et al. 2009), thereby disrupting the “oxidative stress‐inflammation” cycle that drives stone formation.

Interestingly, our MR analysis revealed a site‐specific protective effect of coffee on urinary stones. Unsweetened coffee was linked to a lower risk of upper tract stones, while coffee with sugar was associated with reduced lower tract stone risk. However, after adjusting for confounders like hypertension, T2DM, BMI, and smoking in MVMR models, the protective effect of sugary coffee was no longer observed. These differences may stem from both biological and behavioral factors—unsweetened coffee offers metabolic benefits, while added sugar may diminish them. This highlights the importance of considering sugar intake and metabolic health in dietary studies of stone risk.

Although our MR analysis suggested a protective link between decaffeinated and ground coffee and upper urinary tract stones, this result should be interpreted with caution due to the small number of IV (only 3 SNPs). Despite adequate instrument strength (*F*‐statistics > 10), the limited SNPs reduce the robustness and reliability of the findings (Burgess and Thompson 2011). Specifically, a limited number of SNPs reduces the ability to perform comprehensive sensitivity analyses that are essential for assessing potential horizontal pleiotropy and validating causal estimates (Hemani, Bowden, and Davey Smith 2018). Given these limitations, the observed association should be viewed as hypothesis‐generating. Future studies with larger GWAS datasets and more valid genetic instruments are needed to replicate and validate these findings.

The protective effects of tea consumption against urolithiasis are primarily mediated by its high polyphenol content (e.g., epigallocatechin‐3‐gallate, EGCG, and theaflavins) and modulation of oxalate metabolism via gut microbiota interactions. EGCG, the predominant catechin in green tea, can reduce the crystal‐cell adhesion of calcium oxalate monohydrate (Fong‐Ngern et al. 2017; Kanlaya et al. 2024). Tea‐derived xanthines (such as caffeine and theophylline) induce mild diuresis. Furthermore, tea polyphenols enhance intestinal *Oxalobacter formigenes* colonization, increasing oxalate degradation and reducing its urinary excretion (Daniel et al. 2021).

Psychoactive drinks, including coffee, tea, and cocoa‐based beverages with compounds like caffeine and theobromine, have shown a protective effect against upper urinary tract stones. This association remained significant after MVMR analysis, supporting the protective role of these drinks in nephrolithiasis prevention.

Our study suggested a negative causal relationship between alcohol, white wine, and upper urinary tract stones. A number of previous studies have had inconsistent findings regarding the relationship between alcohol intake and UC (Wei et al. 2024; Zhou et al. 2023). Recently, a large cohort study in China suggested that consumption of ≥ 30.0 g of pure alcohol per day was associated with a reduced risk of kidney stones (Littlejohns et al. 2020). Mechanistically, alcohol suppresses antidiuretic hormone (ADH), increasing urinary output. However, its lithoprotective effects vary by dose and beverage type, with excessive intake paradoxically raising risk through hypercalciuria and hyperuricosuria (Fukui et al. 2023). This relationship is further modulated by beverage type; for example, the protective extent of the same dosage of wine and beer against calculi is different (Shringi et al. 2024), and the protective effect of highly distilled spirits is weaker.

Our study confirmed the protective effect of fruit against UC. Several previous studies have suggested that the preventive effect of fresh fruits on kidney stones may benefit from dietary alkali and citrate supplementation (Ferraro et al. 2020; Siener et al. 2003). Citrate is converted to bicarbonate in the liver, which limits its renal reabsorption and increases urinary excretion (Hamm 1990; Simpson 1983). In urine, citrate inhibits calcium oxalate crystal formation and lowers calcium levels by forming calcium citrate (Rimer et al. 2019). Importantly, fruits like plum and cherry are rich in antioxidants, especially polyphenols and ascorbic acid, reduce oxalate‐induced ROS, easing oxidative stress and offering anti‐inflammatory, diuretic, and antibacterial benefits (Gan et al. 2024; Wijayabahu et al. 2019); their role in inhibiting stones was further emphasized.

In particular, our study also suggested a protective effect of dried fruit against upper urinary tract stones. Despite the indication of horizontal pleiotropy in the initial two‐sample MR analysis, the association between dried fruit consumption and upper urinary tract stones remained robust in MVMR analysis after adjusting for key lifestyle and metabolic risk factors; it points to a potential independent role of dried fruit. As concentrated forms of whole fruits, dried fruits retain high levels of beneficial bioactive compounds, including citric acid and polyphenols, potentially enhancing their antioxidative and alkalinizing effects on the urinary tract.

Additionally, our mediation analysis indicates that calcium may partially mediate the protective effects of fruit, dried fruit, and cherry on upper urinary tract stones. These fruits are rich in potassium, magnesium, and citric acid, which help regulate calcium balance and reduce urinary calcium crystallization. Although calcium accounts for only a small portion (2.6%–8.8%) of the total effect, this modest mediation is still biologically plausible. It suggests that while calcium‐related pathways may contribute to the reduction of stone risk, they are not the sole explanation. Other bioactive compounds—like antioxidants, fiber, and organic acids—may also help regulate urine composition and prevent crystal formation. Thus, the limited mediating role of calcium does not diminish its relevance but rather highlights the multifactorial nature of the protective effects exerted by fruit consumption. suggesting that other mechanisms also contribute to the protective role of fruit intake against urolithiasis.

Although previous observational studies have suggested that dairy intake may reduce urolithiasis risk (Asoudeh et al. 2022; Yang et al. 2024), our MR analysis found no significant causal link between dairy‐related factors and UC. This may be due to the lifelong nature of MR estimates, weak or nonspecific genetic instruments, and the complexity of dietary patterns. These null results do not rule out a potential role of dairy or animal protein but highlight the need for more precise dietary measures and stronger genetic tools in future research.

Notably, all dietary exposures exhibited *F*‐statistics below 10 in the MVMR results, indicating a potential risk of weak instrument bias. This may be attributable to the limited number and modest effect sizes of genetic variants associated with specific dietary traits, as well as potential collinearity among exposures in the multivariable model. While these limitations warrant cautious interpretation, the consistency of findings across two‐sample MR analyses provides some support for the robustness of the observed associations. Future research could reinforce these findings by incorporating stronger instruments, increasing sample size, or applying alternative strategies such as Bayesian MR methods (Zuber et al. 2020).

The main strength of this study is its MR design, which strengthens the causal relationship between dietary intakes and UC. Moreover, stratifying urinary stones into upper and lower tract types offers a clearer view of site‐specific dietary effects. Using the updated FinnGen Consortium (R12) database, we obtained reliable UC data. A total of 225 dietary exposures were analyzed, providing strong evidence on the potential associations and causal links between diet and urolithiasis risk. MVMR helped minimize confounding influences, while two‐step MR analyses suggested that serum calcium may partially mediate the protective effects of certain fruits on stone formation, offering insights for future research. However, as the population included in this study comprised only individuals of European ancestry, this may result in population bias that limits the generality of the study to populations of other ancestry. We did not stratify UC by stone composition (e.g., calcium oxalate, uric acid, or struvite), which may have obscured subtype‐specific dietary associations. Some MR results showed heterogeneity, but we did not conduct detailed sensitivity analyses to explore its sources. Additionally, the small number of available SNPs in some analyses (e.g., only three SNPs) may have reduced statistical power and reliability, increasing susceptibility to weak instrument bias. Further research is needed to clarify the biological mechanisms underlying these associations.

## 5 Conclusion

In conclusion, our MR analysis confirmed that coffee, tea, alcohol, psychoactive beverages, fruits, and dried fruit are protective factors for UC. After conducting multivariable Mendelian randomization (MVMR) analyses, the protective effects of several types of coffee, fruits, and psychoactive beverages on UC remained robust, suggesting their independent roles in disease prevention. Moreover, calcium plays a minor mediating role in the protective effect of fruit on UC, which provides new ideas for the future prevention of UC and the development of therapeutic interventions.

## Author Contributions

**Youjia Qiu:** conceptualization (equal), formal analysis (equal), writing – original draft (equal). **Xinling Tang:** conceptualization (equal), formal analysis (equal), writing – original draft (equal). **Bingyi Song:** data curation (equal). **Yuchen Tao:** data curation (equal). **Ziqian Yin:** methodology (equal), software (equal). **Menghan Wang:** methodology (equal), software (equal). **Na Ji:** methodology (equal), software (equal). **Zhouqing Chen:** supervision (equal), validation (equal), writing – review and editing (equal). **Zhong Wang:** funding acquisition (equal), resources (equal), supervision (equal), validation (equal), writing – review and editing (equal). **Xuedong Wei:** funding acquisition (equal), resources (equal), supervision (equal), validation (equal), writing – review and editing (equal).

## Ethics Statement

The authors have nothing to report.

## Conflicts of Interest

The authors declare no conflicts of interest.

## Supporting information


**Figure S1:** fsn370958‐sup‐0001‐FiguresS1‐S8.docx.

**Table S1:** fsn370958‐sup‐0002‐TablesS1‐S16.xlsx.

## Data Availability

The genetic association summary statistics used in this study are all publicly available. Summary‐level data for dietary factors were obtained from two published studies. Summary‐level data for urinary stone disease (included calculus of kidney and ureter and calculus of lower urinary tract) were obtained from FinnGen R12 registry data. Summary‐level data for 30 blood biomarkers were obtained from the IEU OpenGWAS database. All data generated or analyzed during this study are included in the Supporting Information.
