# Supplementary material for: Novel Insights Into the Causal Association Between Dietary Factors and Risk of Urinary Calculus: A Multivariate and Two‐Step Mendelian Randomization Analysis
Source: Food Sci Nutr. 2025 Sep 12;13(9):e70958. doi: 10.1002/fsn3.70958 (PMC12426897; doi:10.1002/fsn3.70958)
Supplement: Supplementary file 1 — Figure S1: fsn370958‐sup‐0001‐FiguresS1‐S8.docx. [file FSN3-13-e70958-s001.docx]

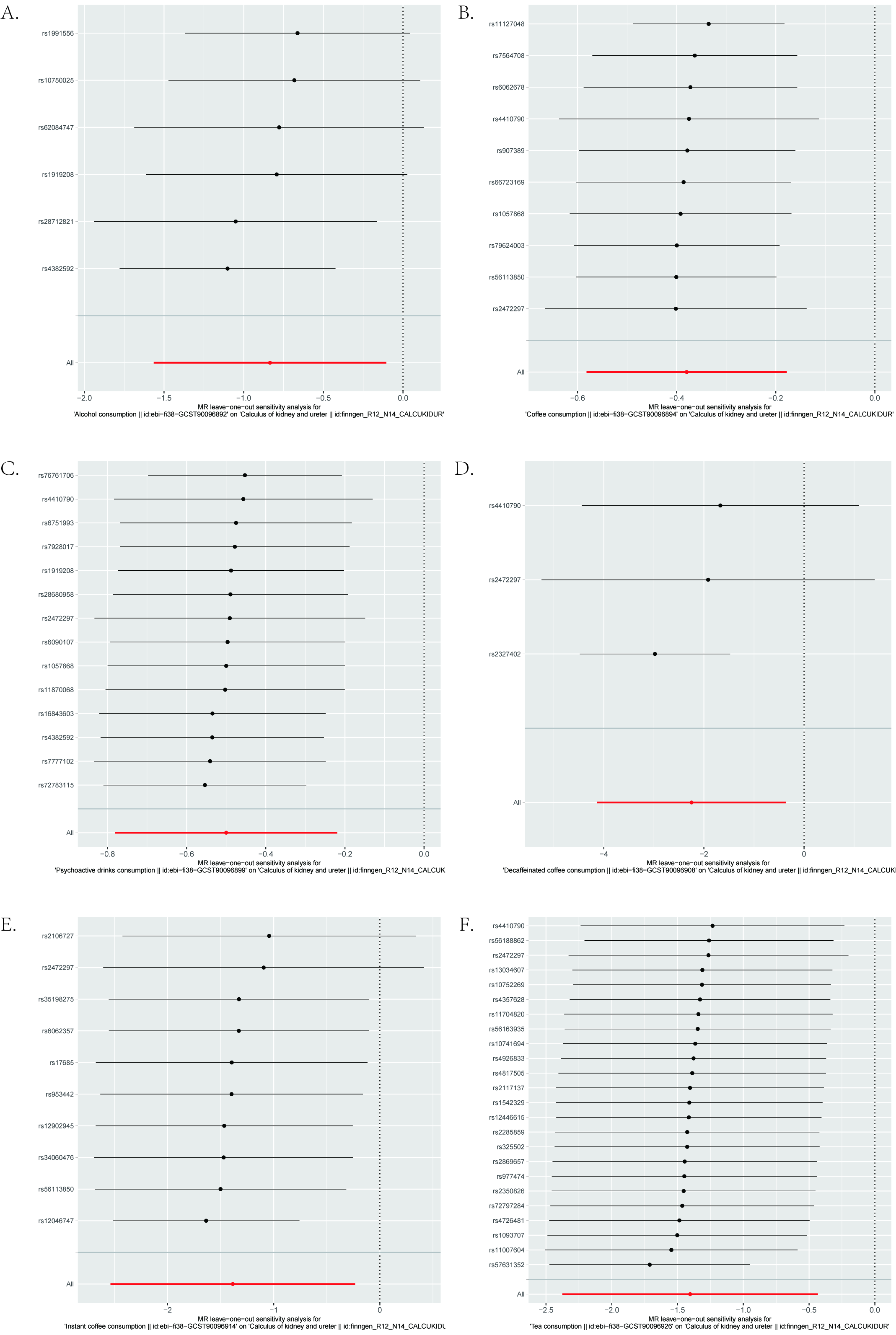


Figure S1-1. Leave-one-out plot of significant dietary factors and calculus of kidney and ureter. (A) Alcohol consumption; (B) Coffee consumption; (C) Psychoactive drinks consumption; (D) Decaffeinated coffee consumption; (E) Instant coffee consumption; (F) Tea consumption.


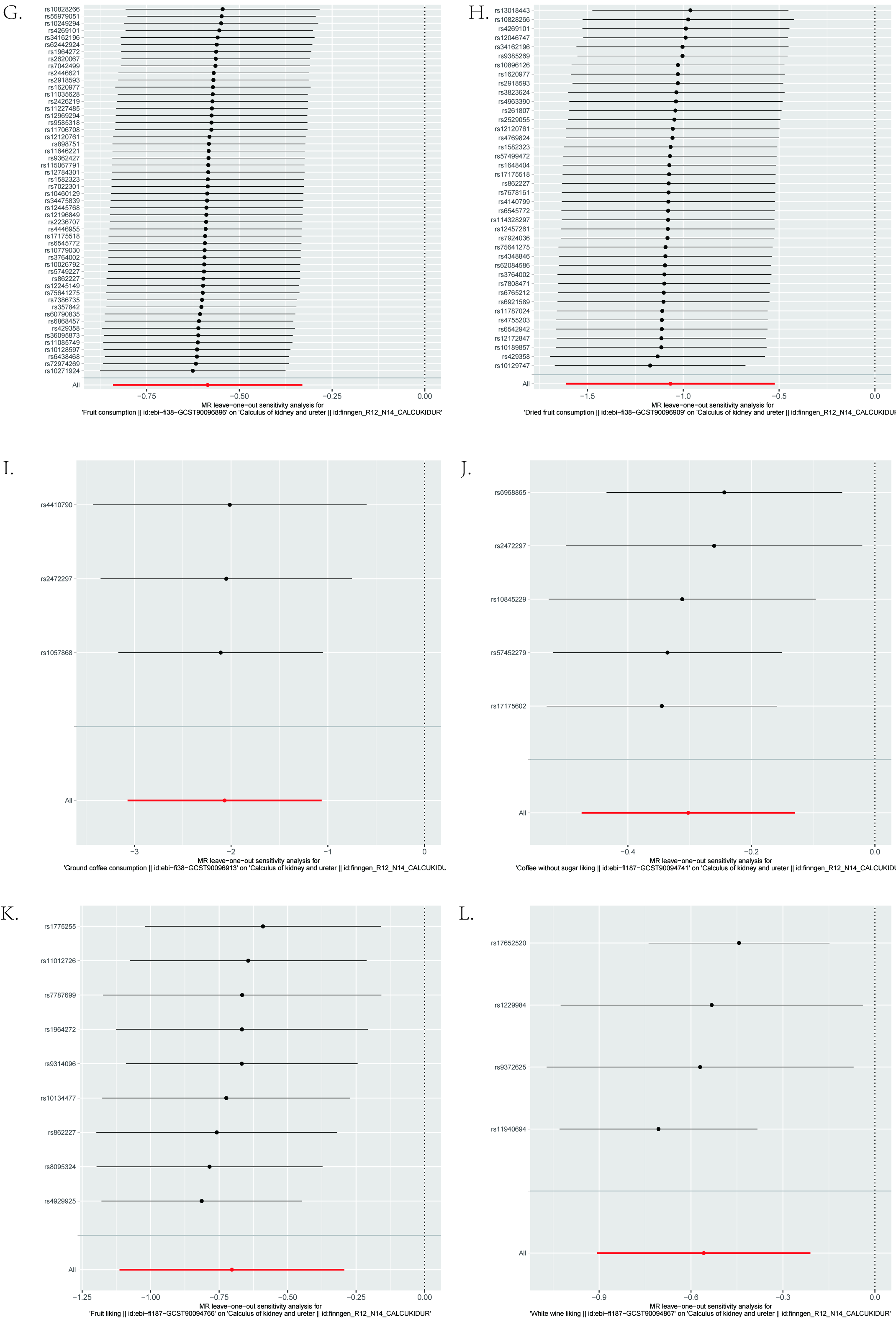


Figure S1-2. Leave-one-out plot of significant dietary factors and calculus of kidney and ureter. (G) Fruit consumption; (H) Dried fruit consumption; (I) Ground coffee consumption; (J) Coffee without sugar liking; (K) Fruit liking; (L) White wine liking.


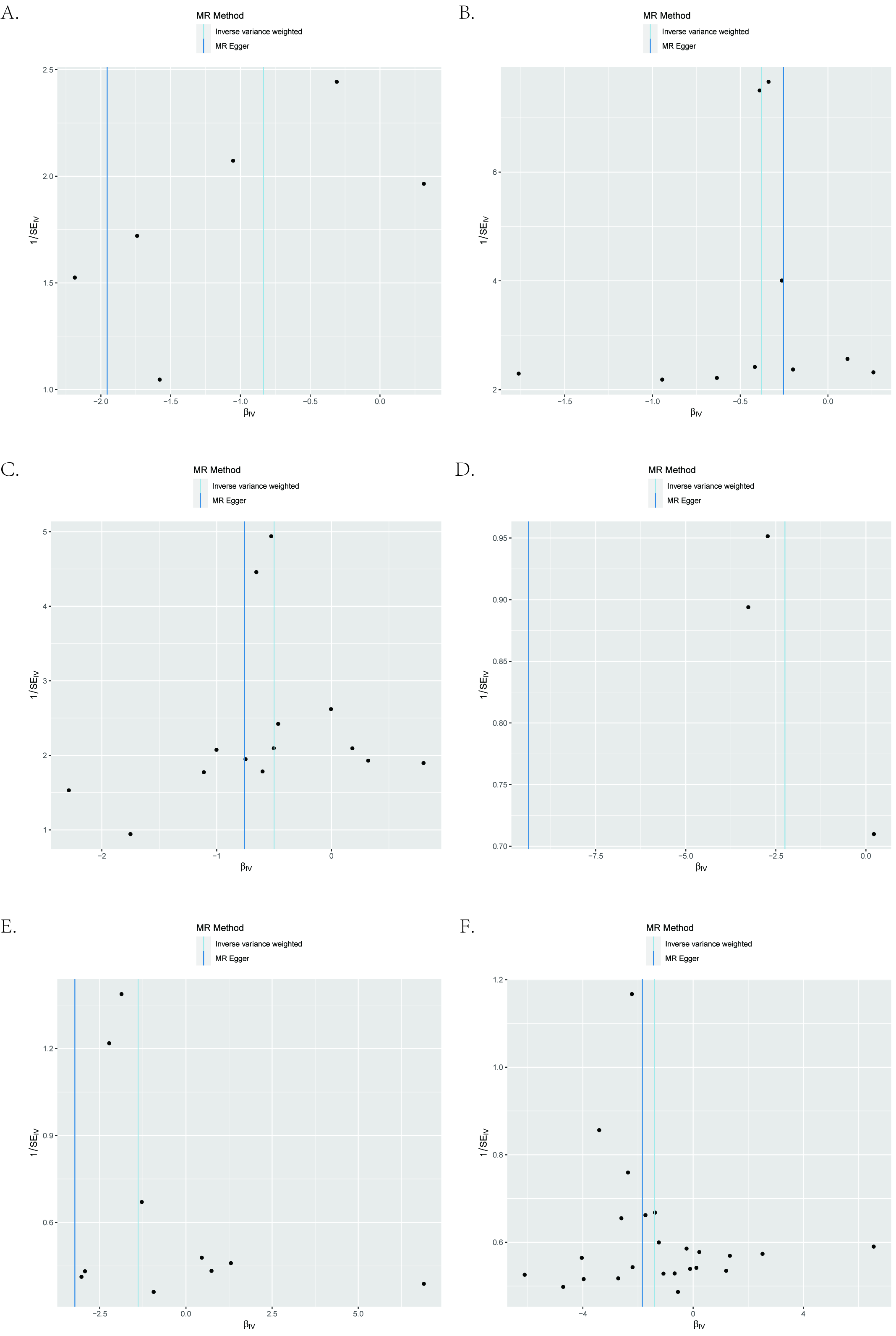


Figure S2-1. Funnel plot of significant dietary factors and calculus of kidney and ureter. (A) Alcohol consumption; (B) Coffee consumption; (C) Psychoactive drinks consumption; (D) Decaffeinated coffee consumption; (E) Instant coffee consumption; (F) Tea consumption.


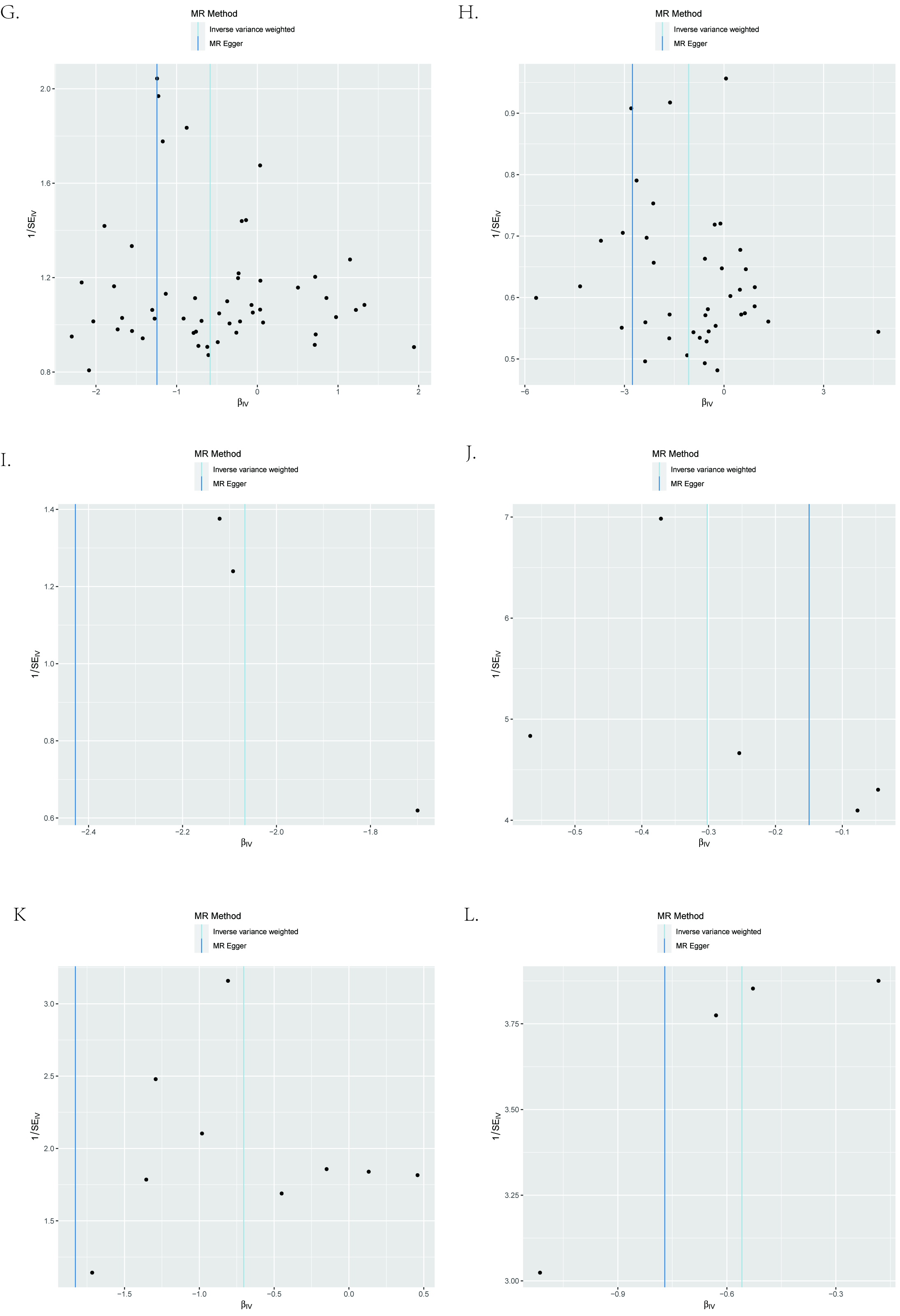


Figure S2-2. Funnel plot of significant dietary factors and calculus of kidney and ureter. (G) Fruit consumption; (H) Dried fruit consumption; (I) Ground coffee consumption; (J) Coffee without sugar liking; (K) Fruit liking; (L) White wine liking.


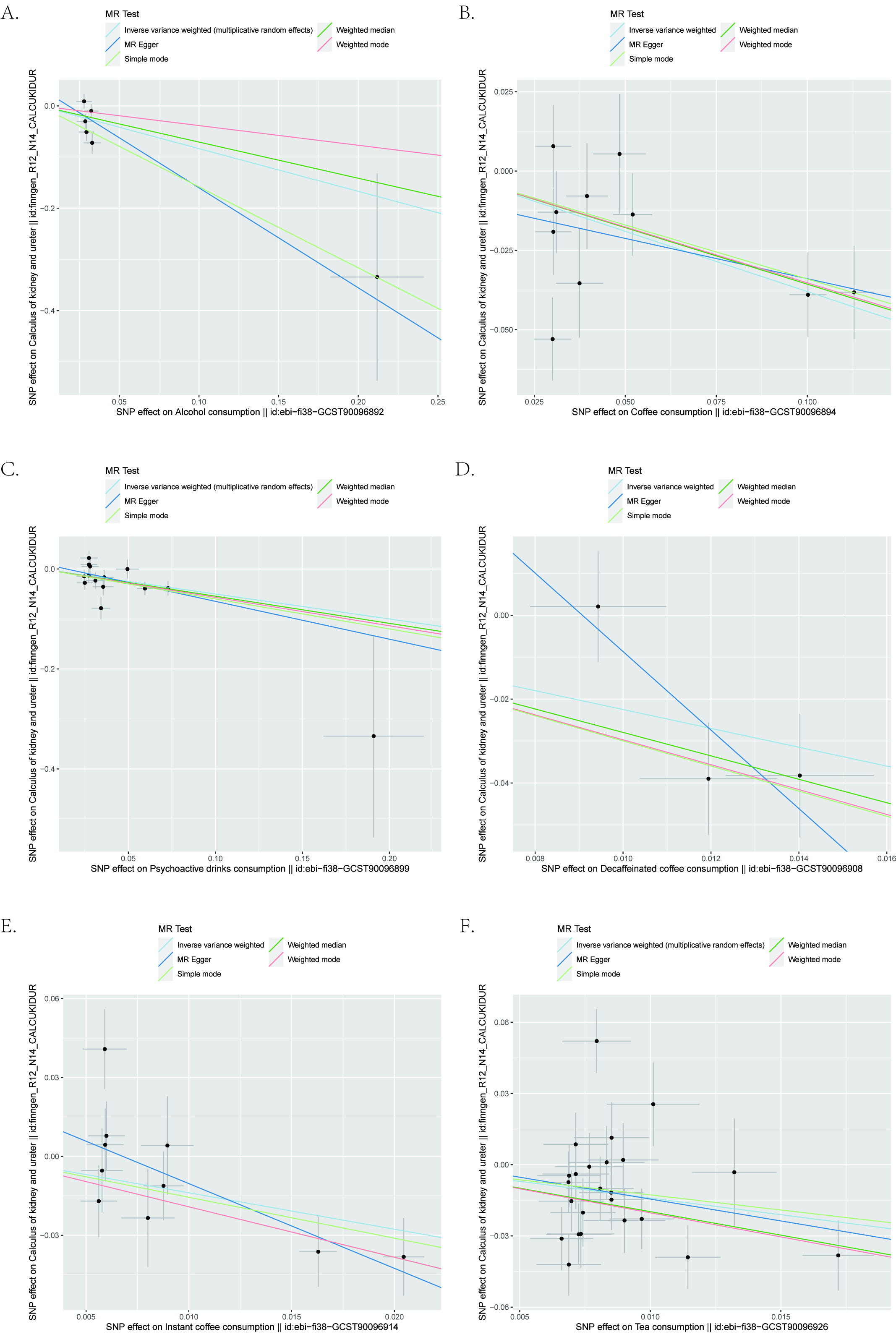


Figure S3-1. Scatter plot of significant dietary factors and calculus of kidney and ureter. (A) Alcohol consumption; (B) Coffee consumption; (C) Psychoactive drinks consumption; (D) Decaffeinated coffee consumption; (E) Instant coffee consumption; (F) Tea consumption.


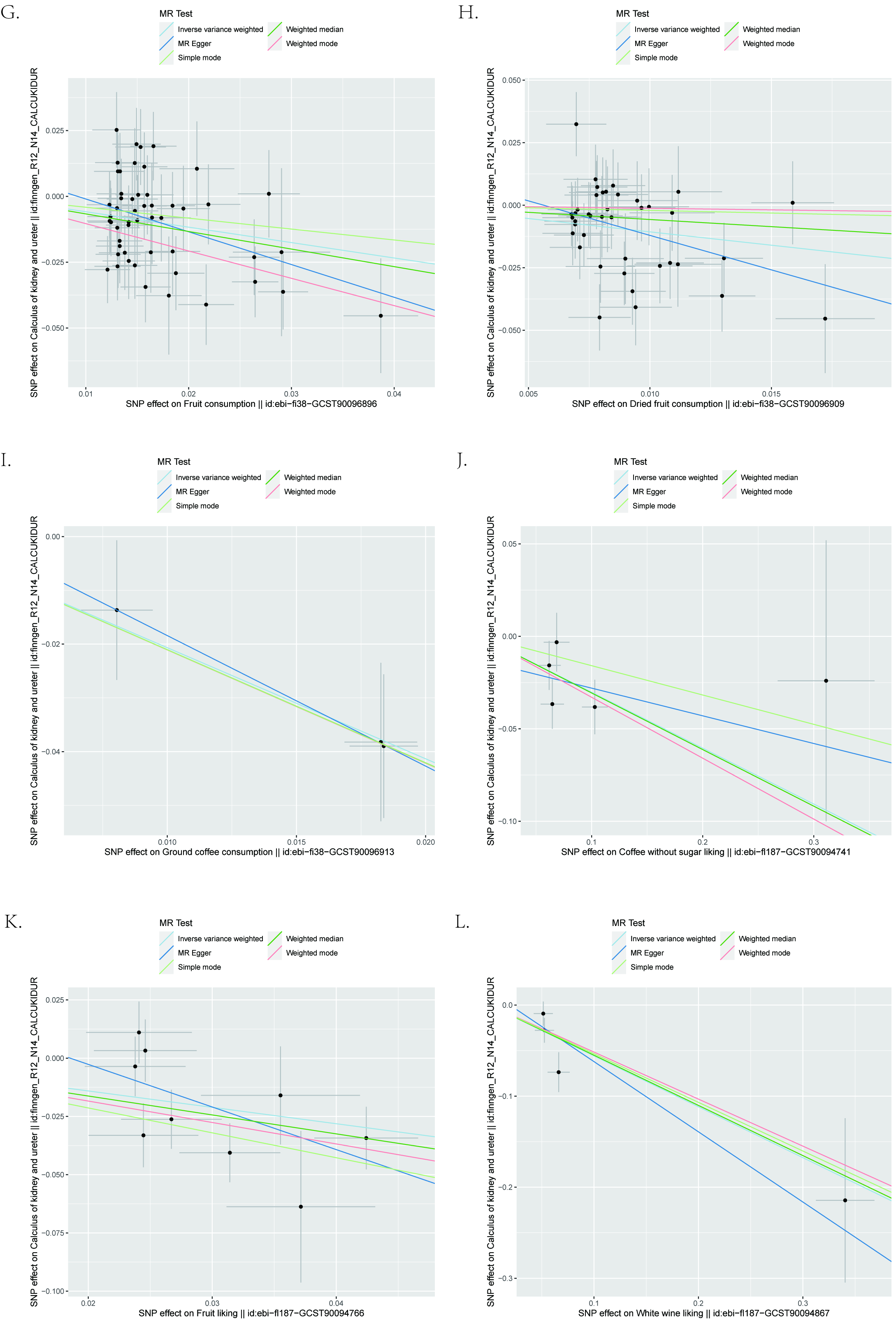


Figure S3-2. Scatter plot of significant dietary factors and calculus of kidney and ureter. (G) Fruit consumption; (H) Dried fruit consumption; (I) Ground coffee consumption; (J) Coffee without sugar liking; (K) Fruit liking; (L) White wine liking.


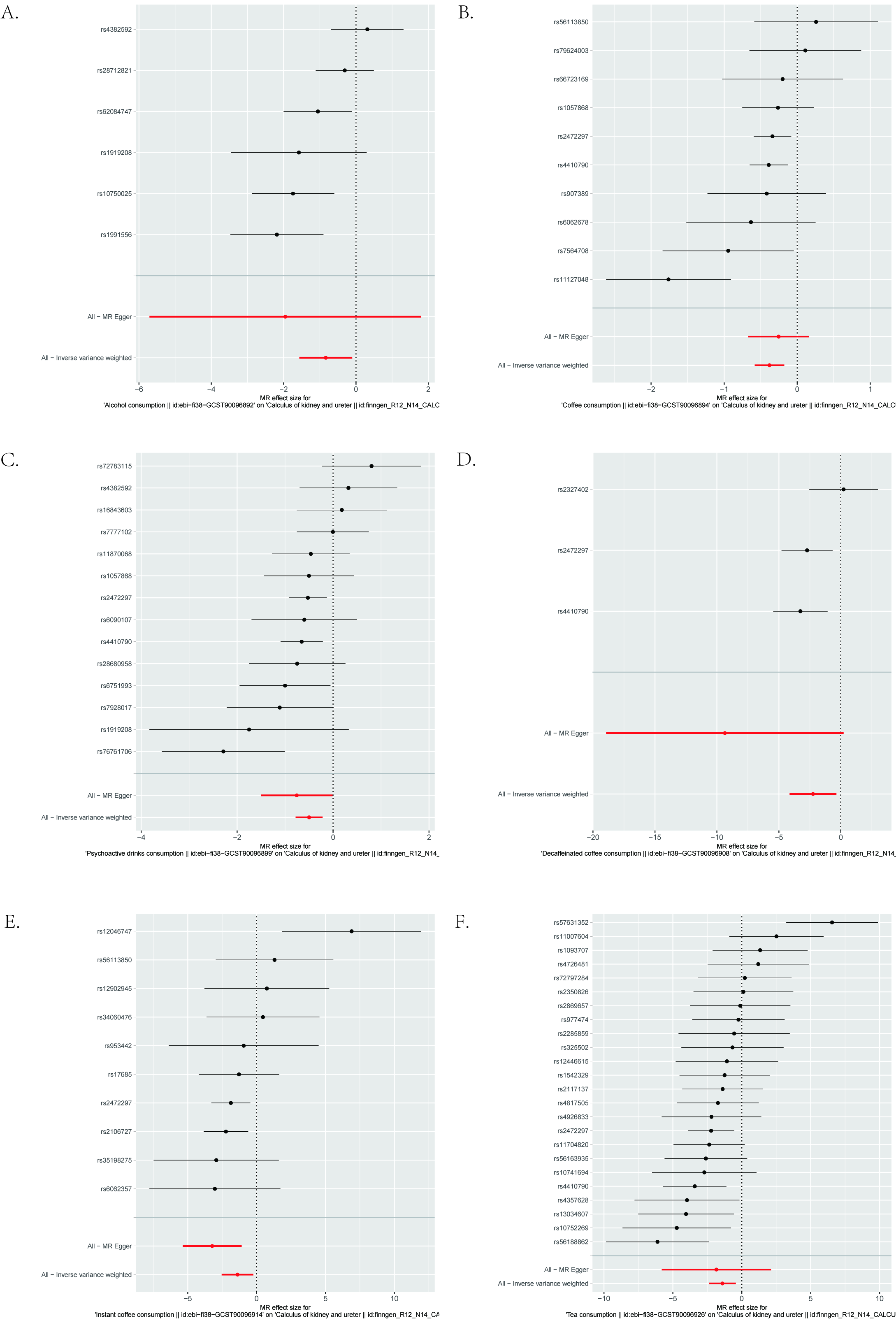


Figure S4-1. Forest plot of significant dietary factors and calculus of kidney and ureter. (A) Alcohol consumption; (B) Coffee consumption; (C) Psychoactive drinks consumption; (D) Decaffeinated coffee consumption; (E) Instant coffee consumption; (F) Tea consumption.


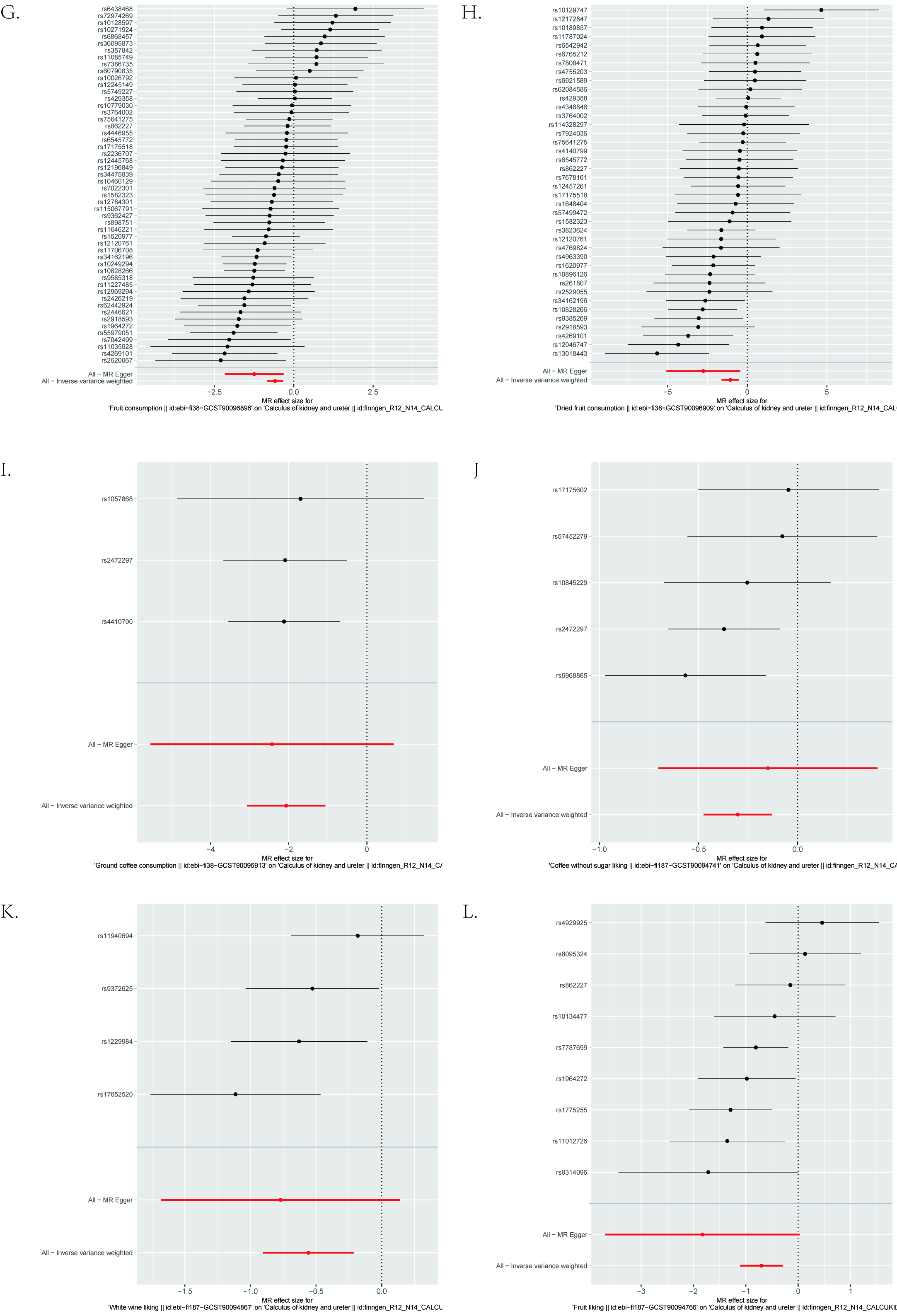


Figure S4-2. Forest plot of significant dietary factors and calculus of kidney and ureter. (G) Fruit consumption; (H) Dried fruit consumption; (I) Ground coffee consumption; (J) Coffee without sugar liking; (K) Fruit liking; (L) White wine liking.


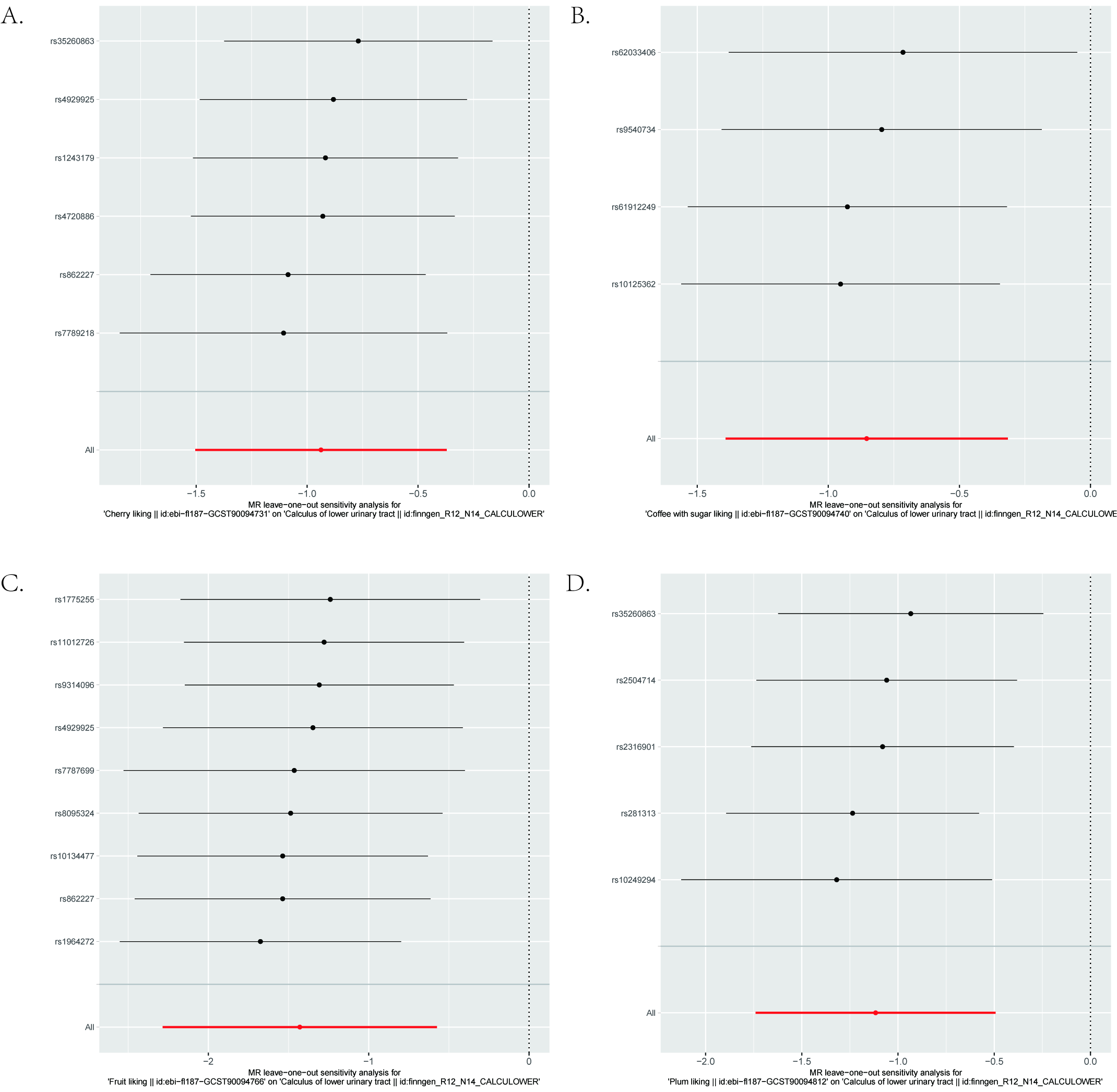


Figure S5. Leave-one-out plot of significan dietary factors of calculus of lower urinary tract. (A) Cherry liking; (B) Coffee with sugar liking; (C) Fruit liking; (D) Plum liking.


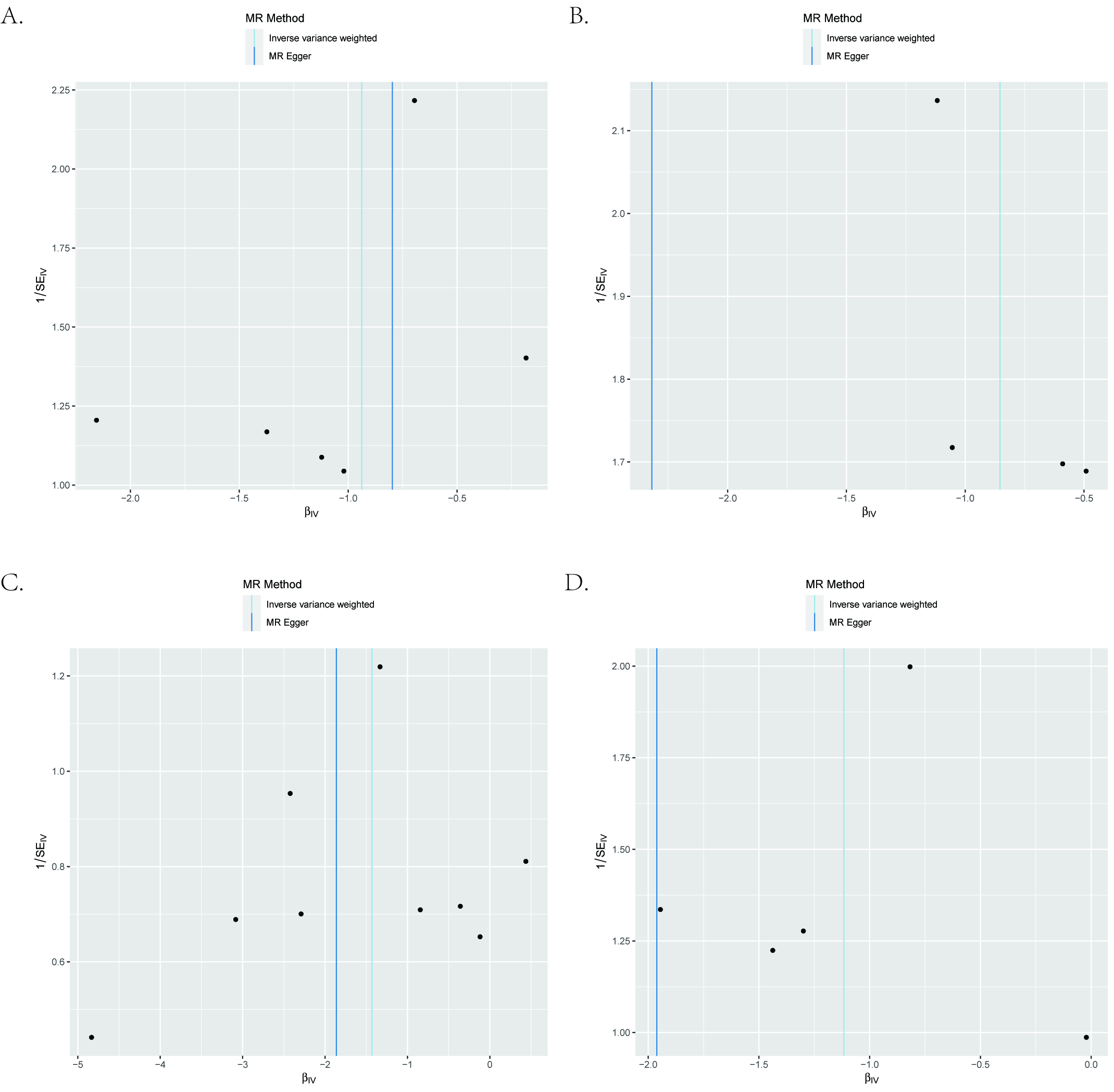


Figure S6. Funnel plot of significant dietary factors of calculus of lower urinary tract. (A) Cherry liking; (B) Coffee with sugar liking; (C) Fruit liking; (D) Plum liking.


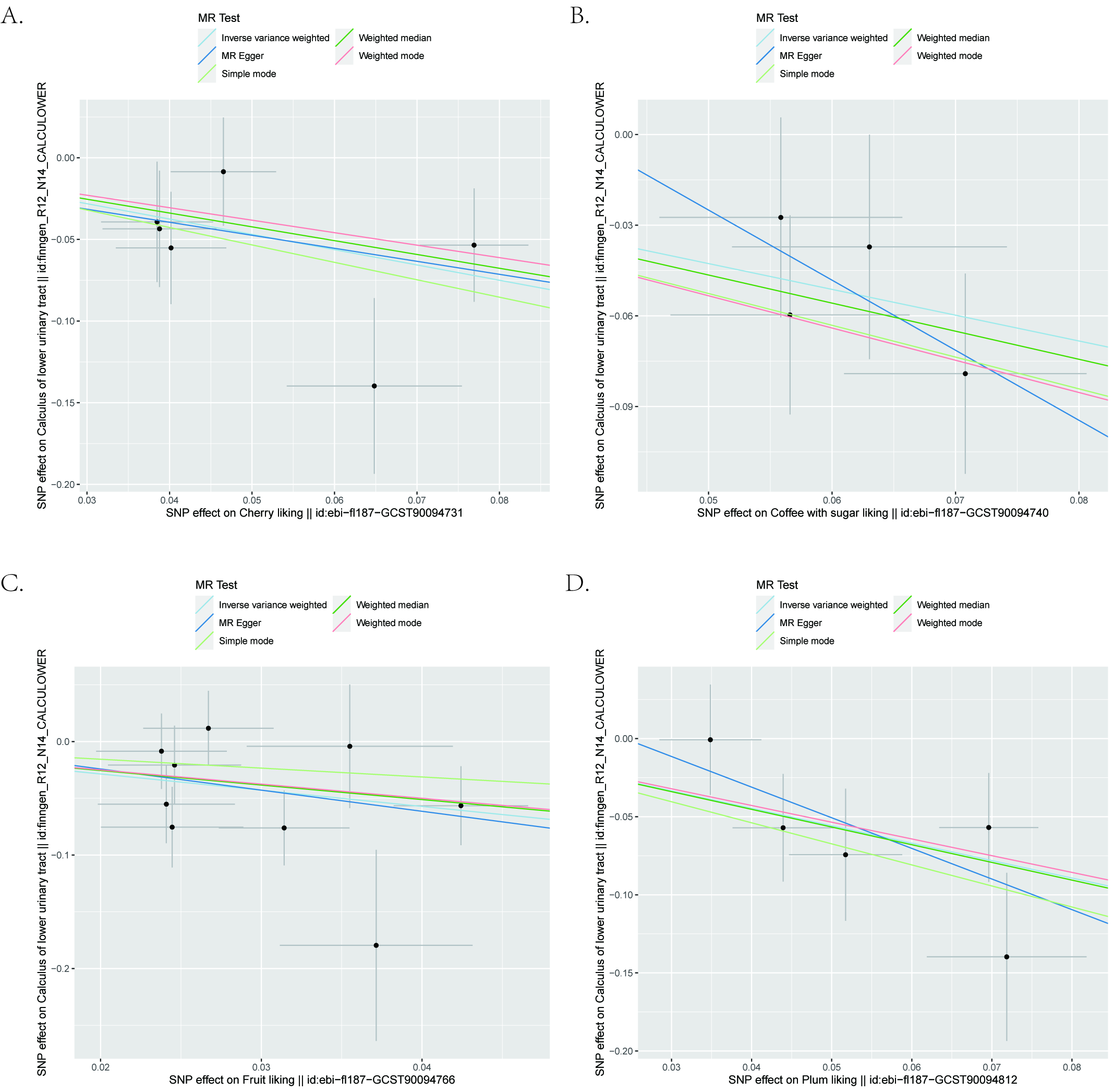


Figure S7. Scatter plot of significant dietary factors of calculus of lower urinary tract. (A) Cherry liking; (B) Coffee with sugar liking; (C) Fruit liking; (D) Plum liking.


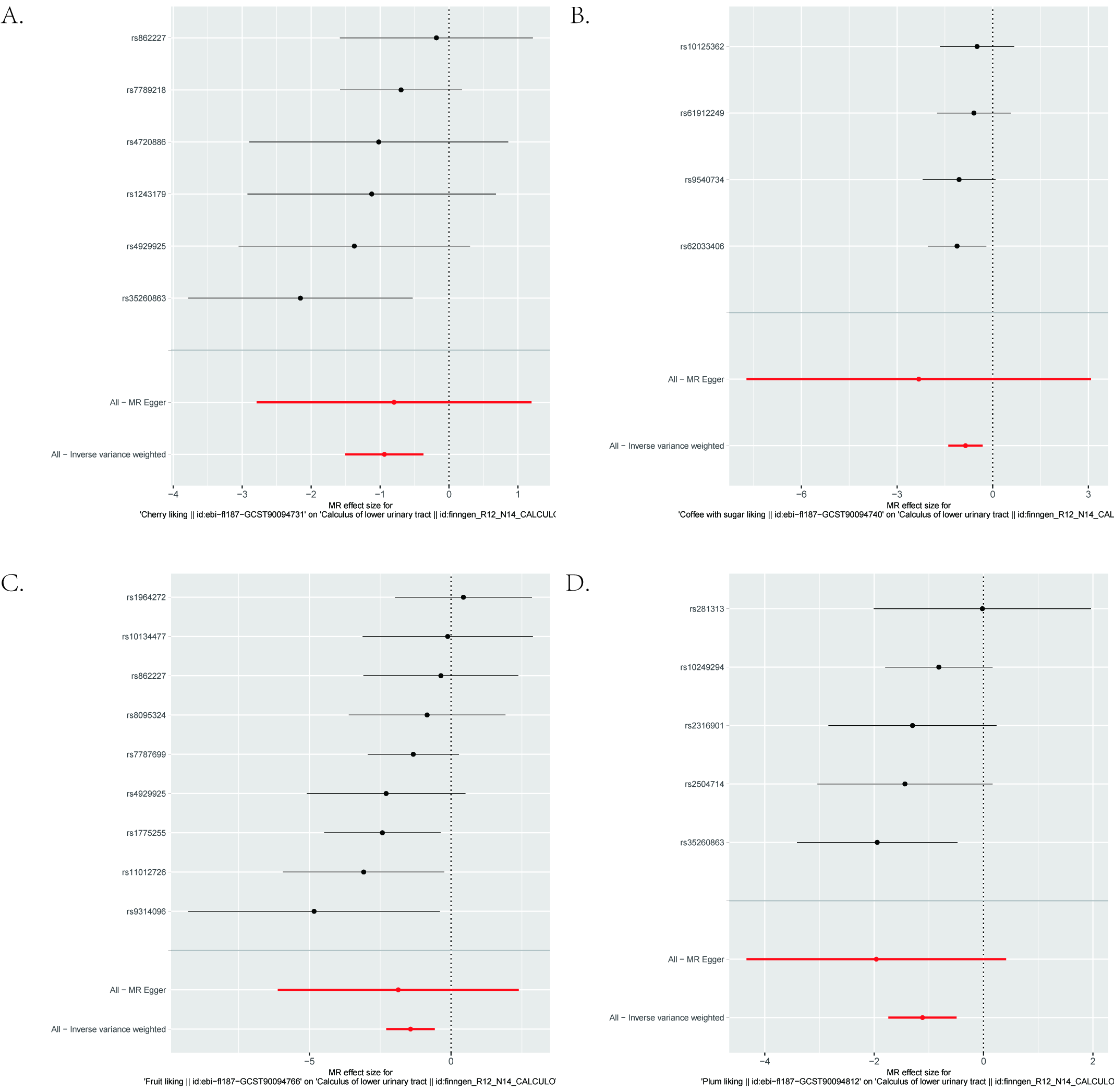


Figure S8. Forest plot of significant dietary factors of calculus of lower urinary tract. (A) Cherry liking; (B) Coffee with sugar liking; (C) Fruit liking; (D) Plum liking.
